# A Case Series of Refractory Pediatric Atopic Dermatitis Effectively Treated With Dupilumab in Combination With Abrocitinib

**DOI:** 10.1111/pde.15761

**Published:** 2024-10-03

**Authors:** Wei Chern Gavin Fong, Htet Hla Win Kaung, Rhea Lopes, Alpa Kanji, Jane Ravenscroft, Ting Seng Tang, Carsten Flohr

**Affiliations:** ^1^ Centre of Evidence Based Dermatology School of Medicine, University of Nottingham Nottingham UK; ^2^ Department of Dermatology Nottingham University Hospitals NHS Trust Nottingham UK; ^3^ Department of Paediatric Dermatology St John's Institute of Dermatology, King's College London and Guy's & St Thomas' NHS Foundation Trust London UK; ^4^ St Thomas' NHS Foundation Trust London UK; ^5^ Paediatric and Population‐Based Dermatology Research St John's Institute of Dermatology, King's College London London UK

**Keywords:** atopic dermatitis, biologics, dupilumab, eczema, Janus kinase inhibitors

## Abstract

Children with severe atopic dermatitis (AD), refractory to conventional systemic treatment as well as single‐agent biologic and Janus kinase inhibitor (JAKi) such as abrocitinib, currently face a lack of treatment options. In response to this clinical conundrum, we present three cases of severe and refractory pediatric AD successfully managed with combined dupilumab and abrocitinib. These children had exhausted all conventional treatments and had undergone treatment with both dupilumab and abrocitinib individually, as well as dupilumab in conjunction with methotrexate. It was only when the combination of dupilumab and abrocitinib was introduced that they finally achieved noticeable and sustained improvements in disease control.

## 1 Introduction

Atopic dermatitis (AD) is a pruritic and inflammatory skin disorder commonly observed in childhood. While the majority of pediatric AD cases can be effectively managed through the elimination or reduction of exacerbating factors, good skin care practices, and the use of topical therapies, there exists a subset of patients who continue to experience extensive disease, resulting in significant physical and emotional burdens [1].

Further treatment options include phototherapy, conventional systemic immunosuppressive treatments (methotrexate, cyclosporine, mycophenalate mofetil, azathioprine), biologic treatments, including dupilumab, and most recently Janus kinase inhibitors (JAKi), such as abrocitinib and upadacitinib.

Real‐world data have revealed a subset of patients who remain significantly burdened, despite addressing all available treatment options. Although combination therapy with topical corticosteroids and topical calcineurin inhibitors alongside dupilumab has been well established [2], there is insufficient published evidence on the effectiveness and safety of dupilumab in combination with other systemic agents, especially more novel treatments such as JAKi.

**FIGURE 1:**
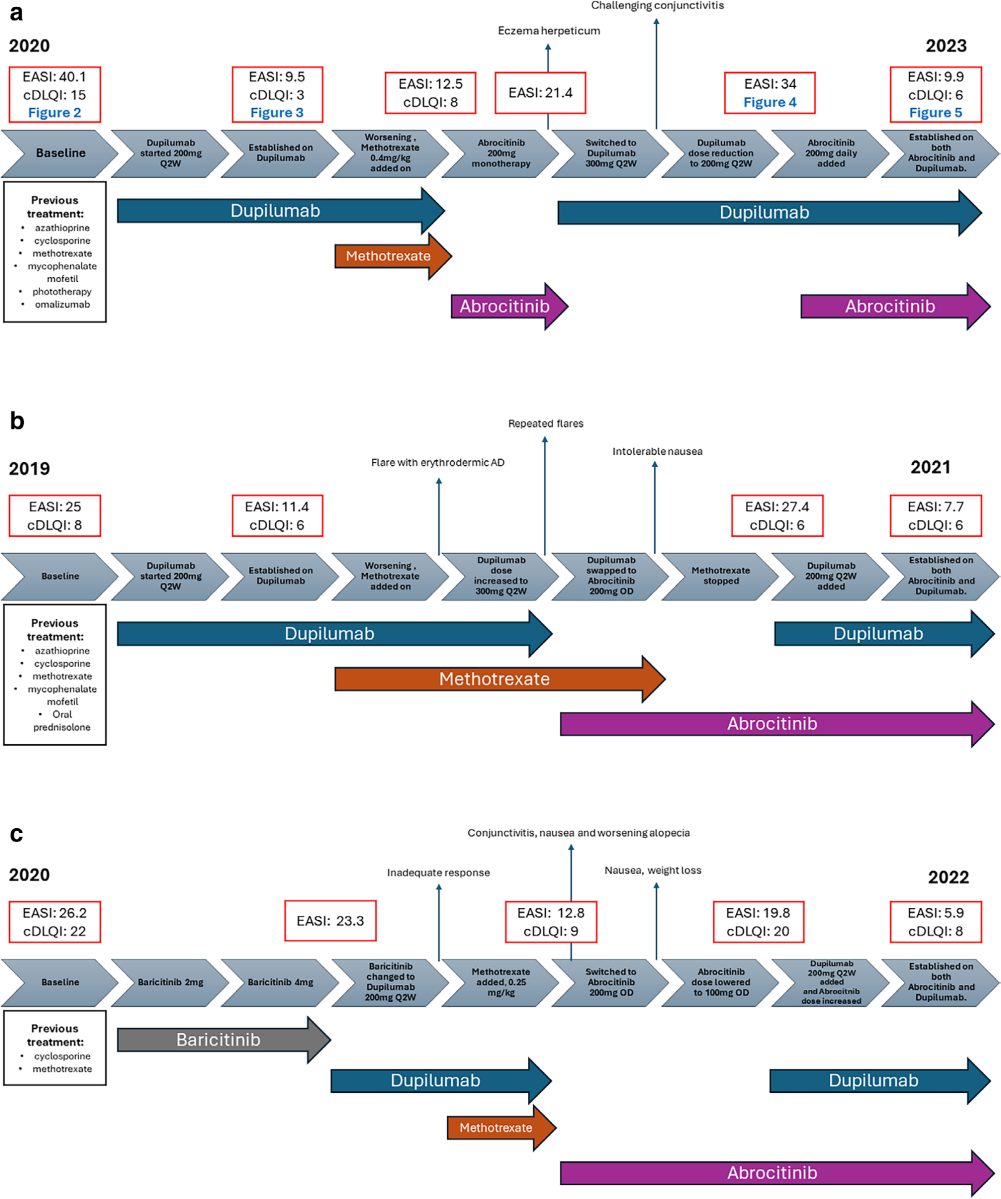
Timelines of treatment for the three cases. (a) Timeline of treatment for Case 1. (b) Timeline of treatment for Case 2. (c) Timeline of treatment for Case 3.

In this article, we present three pediatric cases with severe refractory AD who required combined systemic therapy with dupilumab and abrocitinib to achieve adequate disease control and improvement in quality of life. A summary timeline of all three cases can be found in Figure 1.

## 2 Patient 1

**FIGURE 2:**
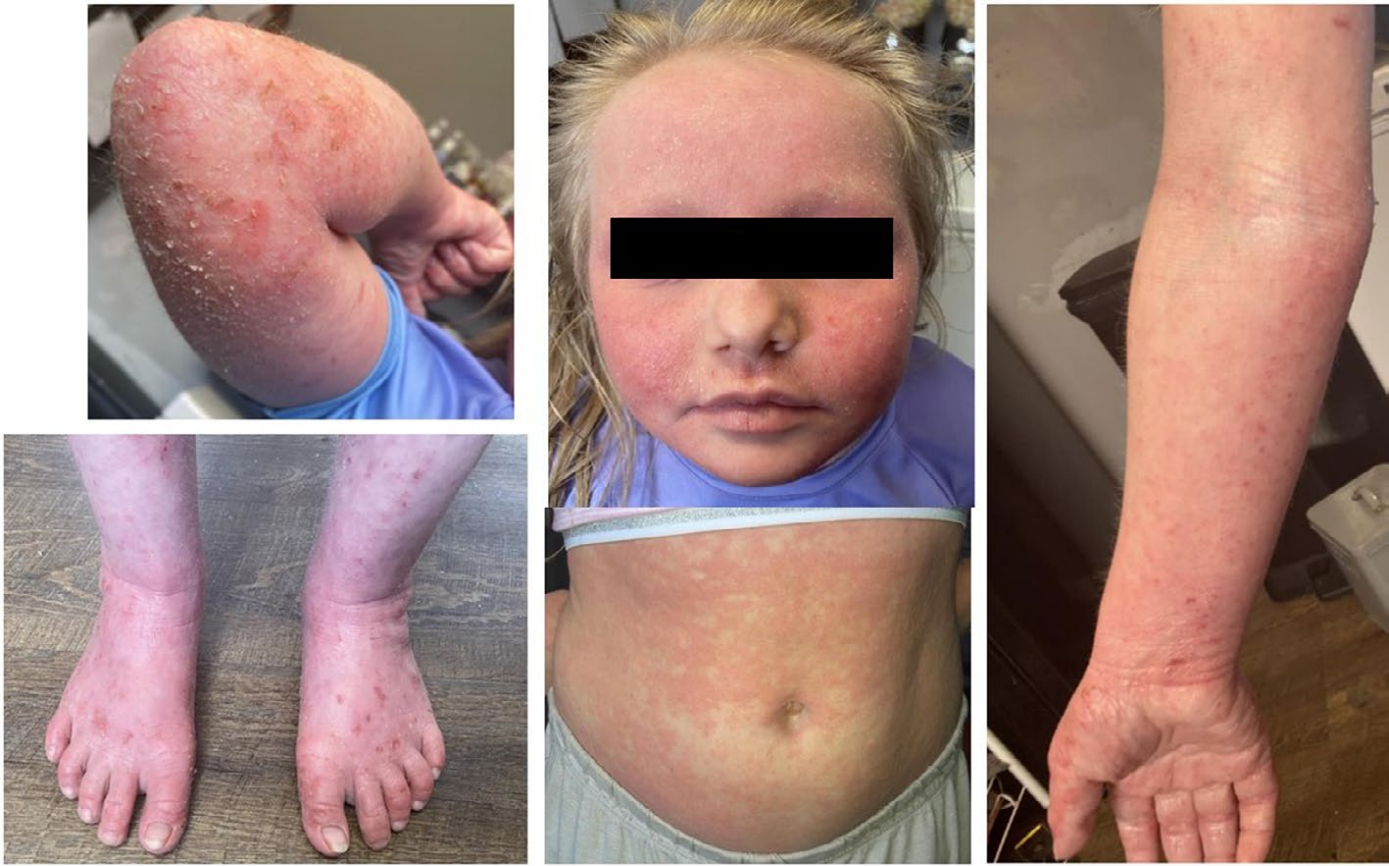
Images of Patient 1 before biologic therapy.

**FIGURE 3:**
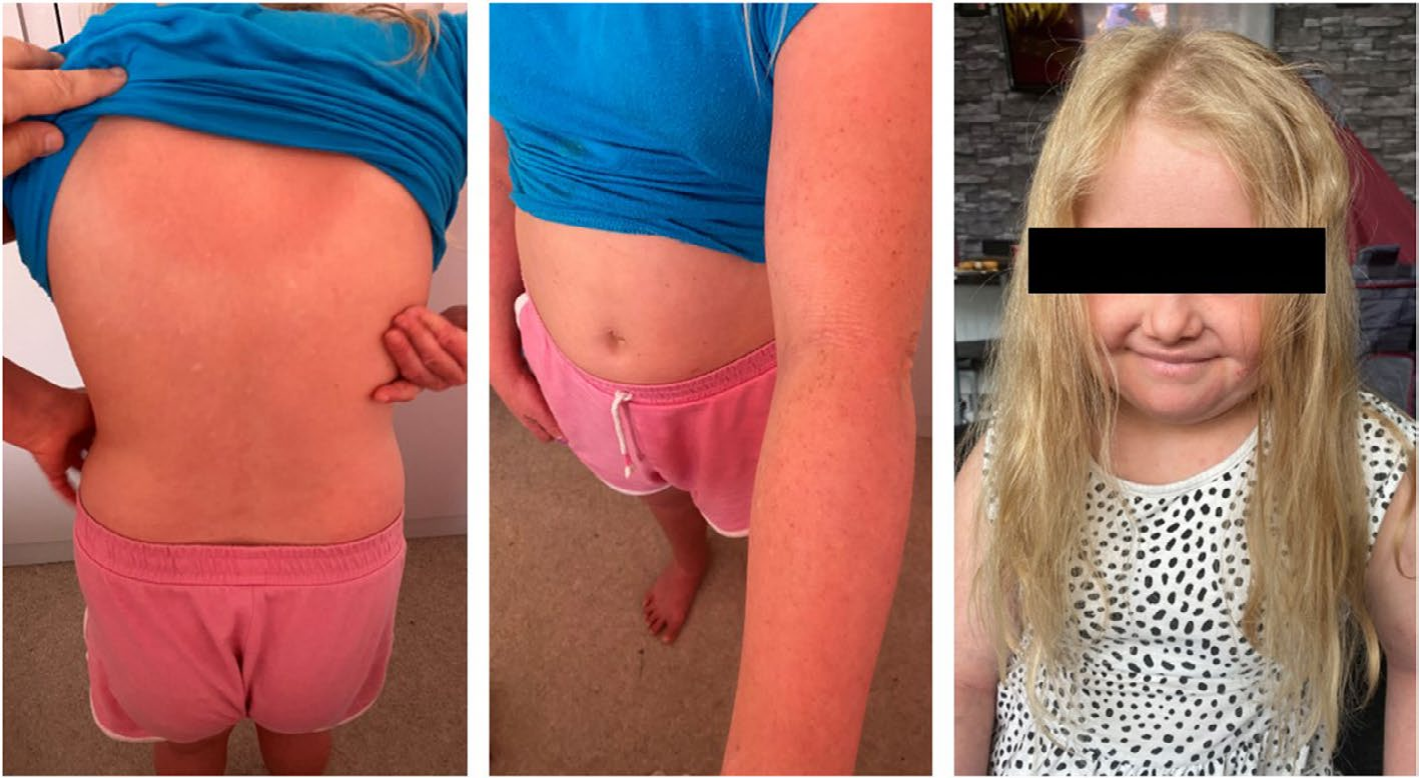
Images of Patient 1 after being established on dupilumab. Please see Figure 1a timeline for corresponding time point.

A 13‐year‐old female with severe, infantile‐onset AD had inadequately responded to conventional systemic therapies (including azathioprine, cyclosporine, methotrexate, mycophenalate mofetil), phototherapy and omalizumab. Her pre‐biologic treatment Eczema Area and Severity Index (EASI) was 40.1 and her Children's Dermatology Life Quality Index (cDLQI) was 15 (Figure 2). She was therefore started on dupilumab 200 mg fortnightly. This initially led to marked improvement in her AD with no adverse effects (EASI 9.5, cDLQI 3) (Figure 3). However, 6 months later, she had a marked disease flare due to a skin infection. Methotrexate was subsequently added at 0.4 mg/kg/week alongside dupilumab as adequate disease control was not recaptured after the flare. This combination of treatment led to only limited additional benefit (EASI 12.5, cDLQI 8).

**FIGURE 4:**
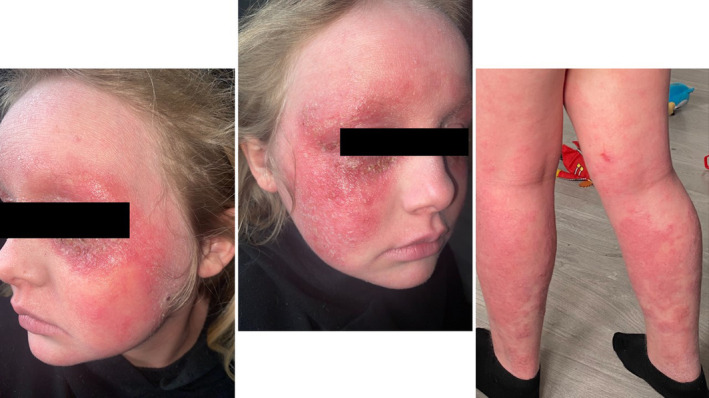
Images of Patient 1 illustrating poorly controlled eczema being on dupilumab monotherapy, following a unsuccessful trial on abrocitinib. Please see Figure 1a timeline for corresponding time point.

She was therefore switched to abrocitinib monotherapy at 200 mg daily. Although there was some improvement in her AD, she had several episodes of herpes simplex virus‐driven exacerbations, including an episode of eczema herpeticum. At this point, her EASI was 21.40. Thus, she was switched back to dupilumab, 300 mg every 2 weeks. Unfortunately, her AD continued to flare, and she also developed challenging conjunctivitis, resulting in a dose reduction to 200 mg every 2 weeks (Figure 4).

**FIGURE 5:**
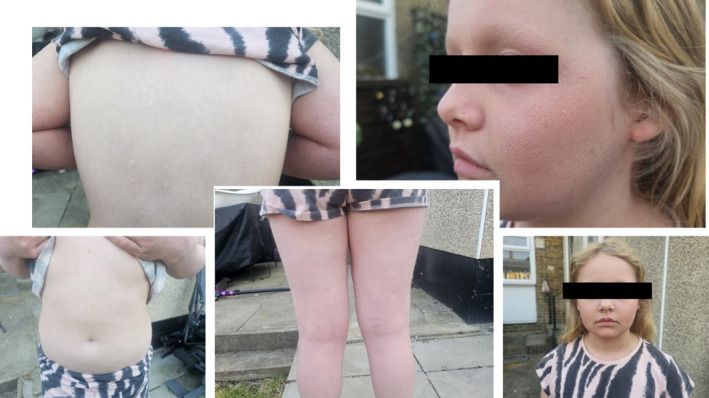
Images of Patient 1 after being established on combination therapy with dupilumab and abrocitinib. Please see Figure 1a timeline for corresponding time point.

Given that her AD remained inadequately controlled on dupilumab 200 mg every 2 weeks, with an EASI score of 34, abrocitinib was added at 200 mg daily. Within a month, there was a significant improvement in her AD, and her conjunctivitis resolved (EASI 9.9, cDLQI 6, 8 weeks after starting abrocitinib). She currently remains well‐controlled 12 months into combined systemic treatment (Figure 5). No further herpetic exacerbations have occurred on antiviral suppressive therapy either. A summary timeline for her case can be found in Figure 1a.

## 3 Patient 2

A 15‐year‐old female with severe childhood‐onset AD had exhausted phototherapy, conventional systemic therapy (including azathioprine, cyclosporine, methotrexate, mycophenalate mofetil, and oral prednisolone) to no avail. Her pre‐biologic EASI was 25, with a patient orientated eczema measure (POEM) of 28, and cDLQI of 8.

In light of this, she was commenced on dupilumab 200 mg every 2 weeks, switching seamlessly from cyclosporine. This initially resulted in some, but not sufficient, improvement (POEM 12, cDLQI 6, and EASI 11.4).

Six months later, subcutaneous methotrexate at 0.3 mg/kg/week was added and then increased to 0.35 mg/kg/week four weeks later to achieve better disease control. Two months later, she was admitted with erythrodermic AD, and thereafter her dupilumab was increased to 300 mg every 2 weeks. Although the patient subsequently developed conjunctivitis, there was overall adequate disease control for several months. Eighteen months later, after several further flares on dupilumab and methotrexate, dupilumab was switched to abrocitinib, 200 mg daily, while maintaining 0.35/kg/week of methotrexate, leading to improved symptoms with no adverse effects.

However, over time, the patient became increasingly nauseous after the methotrexate dose and this was therefore stopped 6 months later. At this point, on abrocitinib alone, her EASI was 27.4, with a POEM of 19, and a cDLQI of 6. Dupilumab 300 mg every 2 weeks was therefore added, alongside abrocitinib 200 mg daily. She has been well‐controlled on this combined systemic treatment strategy since 2021 and she remains well controlled at subsequent follow‐ups (2024 at time of this publication) Her AD has remained stable without major adverse effects. Her latest scores were: EASI 7.7 and cDLQI 6. A summary timeline for her case can be found in Figure 1b.

## 4 Patient 3

A 13‐year‐old male with severe childhood‐onset AD and comorbid alopecia aerata (AA) had been tried on cyclosporine and methotrexate with no improvement in either his AA or AD. His pre‐biologic EASI was 26.2, POEM 16, and cDLQI 22.

He was then entered into a clinical trial of baricitinib for AD. He was randomized to the low dose of 2 mg daily initially for 16 weeks and subsequently increased as per protocol to 4 mg daily. This benefited his AA but not his AD, which was still troublesome, with an EASI score of 23.3. Therefore, after 28 weeks on baricitinib, he was switched to dupilumab 200 mg every 2 weeks. Subsequently, due to limited response, methotrexate was added at 0.25 mg/kg per week orally. At this point, his EASI score was 12.8 and the cDLQI 9.

Unfortunately, on this combination, he experienced worsening conjunctivitis, nausea and worsening AA. He was therefore switched to abrocitinib starting at 200 mg daily. Although there was disease improvement, this was associated with significant nausea, bloating and weight loss, resulting in a reduction in dose to 100 mg daily. Dupilumab was revisited, but refused due to needle phobia.

On the lower dose of abrocitnib, he had a difficult time, with worsening AA, inadequately controlled AD, and resulting anxiety and depression. His EASI was 19.8, POEM was 16 and his cDLQI 20. Thus, abrocitinib was increased back to 200 mg daily and dupilumab 200 mg every 2 weeks was introduced with support from child life specialists for needle phobia. The combination therapy benefited him significantly. Over the last year since starting this combination, his AD has been well‐controlled with minimal topical steroid requirements (EASI 8, cDLQI 8). This was also accompanied by marked improvement in his AA, his confidence, and thus quality of life. A summary timeline for his case can be found in Figure 1c.

## 5 Discussion

To the best of our knowledge, this is the first report describing combined administration of abrocitinib and dupilumab in children with severe, refractory AD.

Children afflicted with severe AD resistant to topical or conventional systemic therapies, may require more novel treatments such as dupilumab. This interleukin (IL)‐4 and IL‐13 inhibitor has extensive clinical trial and real‐world data backing its efficacy and tolerability, with the most common adverse effect being conjunctivitis. It is often considered the first‐line treatment in severe AD that is refractory to conventional systemic treatments. However, it is administered subcutaneously, which may deter needle‐phobic individuals, as illustrated in Patient 3. Recently, abrocinitib and other oral JAKi such as upadacitinib have also been licensed [3] and represent an alternative to biologics. In comparison to the specific interleukin antagonism of biologics, these small molecule treatments exert a broader effect, blocking several signaling pathways in AD‐associated cytokines [4, 5]. Abrocitinib 200 mg once daily has been shown in recent network meta‐analyses to be superior to dupliumab across several indices of efficacy [3, 6]. It is also an oral treatment, which may be more acceptable to children. However, because of its broader mechanism of action, it is associated with increased susceptibility to infections alongside other considerations such as biochemical derangements, increased risk of thrombosis, and potential risk of malignancy. As such, a careful balance of risks versus benefits, alongside shared decision making, needs to be adopted when choosing or switching between these treatments. Independent resources such as the platform eczematherapies.com may assist in this [7].

We also recognize some clinicians may choose to explore cyclosporine as a potential other adjunct systemic medication in addition to dupilumab to achieve AD control in these scenarios. However, cyclosporine is not a drug we would routinely use long term in children, with methotrexate now being more commonly used, especially since the publication of the TREAT trial [8], which showed methotrexate to be a suitable alternative to cyclosporine, both with regard to efficacy but also safety and cost‐effectiveness.

A subset of individuals with severe AD may inadequately respond to monotherapy with dupilumab or JAKis such as abrocitinib, or an initial response may be followed by reduced efficacy, as seen in our patients. Significant clinical improvement and disease stability were observed when abrocitinib and dupilumab therapy were used in combination. This combination strategy was adequately tolerated and appeared to mitigate ophthalmic issues in the first two cases and comorbid AA in the third case.

Our observations align with the work of Yang et al. who recently described the concomitant use of tofacitinib or baricitinib, nonselective JAKis, and dupilumab in adults [9]. They found that significantly more patients treated with tofacitinib or baricitinib in combination with dupilumab achieved EASI‐75 compared to dupilumab monotherapy or dupilumab in combination with another immunosuppressant. Additionally, they similarly showed that this novel combination was well tolerated [9], despite using nonselective JAKis.

The heightened observed efficacy of combined dupilumab and abrocitinib may be attributed to the inhibition of IL‐4, and IL‐13 at two separate stages, at the receptor level (dupilumab) and intracellularly (abrocitinib) (7, 8). Ophthalmic adverse effects associated with dupilumab are postulated to arise from the antagonism of the Th2 pathway leading to the activation of the opposing Th1 pathway [10]. In this context, an additional advantage of this combined regimen is that abrocitinib may attenuate cytokines implicated in this process (6, 7), as seen in Case 1. Furthermore, combination therapy may be particularly appealing if the individual has comorbid AA, as illustrated in Case 3, especially in the UK where it is difficult to access JAKi for AA in children and young people. Methotrexate has been shown in both experimental in vitro [11] and in vivo [12] models to likely inhibit the JAK/STAT pathway. This drug, and its use in conjunction with biologics, is an established clinical practice across various medical disciplines, including dermatology [13]. In rheumatological diseases, this combination has demonstrated greater clinical efficacy compared to utilizing a biologic alone. However, in most countries, methotrexate is used off‐label for AD. Additionally, methotrexate is linked with both inconvenient and potentially significant adverse effects, leading to variable adherence. The children in our study experienced variable and short‐lived clinical improvement on methotrexate alongside dupilumab. Some clinicians may choose to use higher (greater than 0.4 mg/kg/week) doses of methotrexate as an adjunct treatment to dupilumab. We acknowledge this could have been an option in our patients but we decided against it for the following reasons: (1) these children had already previously failed on methotrexate monotherapy; (2) the side effect profile of methotrexate, especially at higher doses; and (3) our treatment decisions were all based on multi‐disciplinary team (MDT) discussions and meetings. In general, we do not use higher than 0.4 mg/kg/week doses in the UK, in keeping with the European treatment guideline for AD [14]. It is on this premise that we introduced abrocitinib, a more targeted inhibitor of JAK compared with methotrexate, alongside dupilumab, yielding enhanced efficacy and tolerability. This superiority of JAKi over methotrexate has also been observed in other specialties [15].

The cost and access to these therapies may present significant challenges. Obtaining access for even one of these agents is difficult, and obtaining coverage for both in combination may be an even more arduous task often involving extensive documentation, persistent follow‐ups, and multidisplinary discussion. Indeed this was our experience for the three cases. Despite these barriers, the potential benefits of combination therapy highlight its potential value and merit further exploration.

In conclusion, the concurrent use of dupilumab with abrocitinib represents a potentially novel, tolerated and effective therapeutic approach for children and young people with severe, refractory AD who exhibit inadequate control with novel/targeted systemic monotherapy or novel/targeted systemic monotherapy in combination with a conventional systemic agent. However, further prospective studies are warranted to ascertain the long‐term efficacy and safety of this treatment strategy.

## Conflicts of Interest

C.F. is Chief Investigator of the UK‐Irish Atopic eczema Systemic Therapy Register (A‐STAR; ISRCTN11210918) and a Principle Investigator in the European Union (EU) Horizon 2020‐funded BIOMAP Consortium (http://www.biomap‐imi.eu/). He also leads the EU Trans‐Foods consortium and is Director of the Global Atopic Dermatitis Atlas (GADA). His department has received funding from Sanofi‐Genzyme and Pfizer for skin microbiome work. He has also received compensation from the *British Journal of Dermatology* (Reviewer and Section Editor) and EuroGuiDerm (Guidelines Lead). W.C.G.F. owns stocks in Sanofi, GSK, and AstraZeneca. All other authors report no relevant conflicts of interest.

## Data Availability

Data sharing is not applicable to this article as no new data were created or analyzed in this study.
